# Downregulated lncRNA RCPCD promotes differentiation of embryonic stem cells into cardiac pacemaker-like cells by suppressing HCN4 promoter methylation

**DOI:** 10.1038/s41419-021-03949-5

**Published:** 2021-07-02

**Authors:** Ye Zhu, Jia You, Wei Wei, Jianjun Gu, Chao Xu, Xiang Gu

**Affiliations:** 1 Clinical Medical College of Yangzhou University, Yangzhou, China; 2 Department of Cardiology, Northern Jiangsu People’s Hospital, Yangzhou, China; 3 Department of Internal Medicine, Yangzhou Maternal and Child Health Care Hospital, Yangzhou, Jiangsu 225001 China; 4 Department of Biostatistics and Epidemiology, University of Oklahoma Health Science Center, Oklahoma City, OK 73104 US

**Keywords:** Arrhythmias, Stem-cell research

## Abstract

Long non-coding RNA (lncRNA) is receiving increasing attention in embryonic stem cells (ESCs) research. However, the roles of lncRNA in the differentiation of ESCs into pacemaker-like cells are still unclear. Therefore, the present study aims to explore the roles and mechanisms of lncRNA in the differentiation of ESCs into pacemaker-like cells. ESCs were cultured and induced differentiation to pacemaker-like cells. RNA sequencing was used to identify the differential expression lncRNAs during the differentiation of ESCs into pacemaker-like cells. Cell morphology observation, flow cytometry, quantitative real-time polymerase chain reaction, western blot, and immunofluorescence were used to detect the differentiation of ESCs into pacemaker-like cells. LncRNA and genes overexpression or knockdown through transfected adenovirus in the differentiation process. The fluorescence in situ hybridization (FISH) detected the lncRNA location in the differentiated ESCs. Luciferase reporter gene assay, methylation-specific PCR, chromatin immunoprecipitation assay, and RNA immunoprecipitation assay were performed to reveal the mechanism of lncRNA-regulating HCN4 expression. Rescue experiments were used to confirm that lncRNA regulates the differentiation of ESCs into pacemaker-like cells through HCN4. We cultured the ESCs and induced the differentiation of ESCs into pacemaker-like cells successfully. The expression of lncRNA RCPCD was significantly decreased in the differentiation of ESCs into pacemaker-like cells. Overexpression of RCPCD inhibited the differentiation of ESCs into pacemaker-like cells. RCPCD inhibited the expression of HCN4 by increasing HCN4 methylation at the promoter region through DNMT1, DNMT2, and DNMT3. RCPCD inhibited the differentiation of ESCs into pacemaker-like cells by inhibiting the expression of HCN4. Our results confirm the roles and mechanism of lncRNA RCPCD in the differentiation of ESCs into pacemaker-like cells, which could pave the path for the development of a cell-based biological pacemaker.

## Introduction

The sinoatrial node (SAN) is the natural pacemaker of the heart that is composed of pacemaker cells [1]. Sick sinus syndrome (SSS) is usually caused by the disorders of SAN impulse formation or SAN-to-atrial impulse conduction, which comprises a sinoatrial block, sinus bradycardia, and sinus arrest [2]. The increasing implantation rate of electronic pacemakers has been effectively improved sinus node dysfunction. However, electronic pacemakers are limited by potential infections, battery life, electrode wire breakage, and lack of autonomic nervous response [3]. Therefore, it is necessary to develop a biological pacemaker that overcomes the limitations of electronic pacemakers as well as ethical and immunogenic issues, which may be generated from pluripotent stem cells.

The high self-renewal capacity, non-immunogenicity, and plasticity of pluripotent stem cells, which make them an ideal source for regenerative purposes [4]. Previous researches have reported that the ESCs may differentiate into a cardiac pacemaker subtype relevant to embryonic pacemaker development [5]. Angela Scavone et al. have explored the possibility of using CD166 expression for isolating SAN progenitors from differentiating embryonic stem cells (ESCs). They demonstrated that the isolation of cardiac precursors from non-teratogenic populations was able to mature and form a fully functional SAN-like tissue [6]. Stephanie et al. described a transgene-independent method for the generation of SAN-like pacemaker cells from human pluripotent stem cells, with their function as a biological pacemaker in vitro and in mice [7]. The expression of transcription factors has an important role in the differentiation into pacemaker-like cells. Darche et al. [1] revealed that haMSC from human adipose tissue can be partly differentiated to cardiac pacemaker cells. Zhao et al. [2] reported that overexpression of TBX3 and HCN2 could reprogram hiPSC-CMs into pacemaker-like cells. Gorabi et al. [8] found that the TBX18 gene can conduct induced pluripotent stem cells (iPSCs) to differentiate into pacemaker‐like cells. Ionta et al. [5] found that overexpression of SHOX2 during ESC differentiation could upregulate the pacemaker gene program, enhance automaticity in vitro and induce biological pacing upon transplantation in vivo. However, the regulation of transcription factors in differentiation into pacemaker-like cells remains to be elucidated.

Long non-coding RNAs (lncRNAs) are a class of RNAs that are >200 nucleotides in length [9], which are involved in diverse biological processes, such as epigenetic regulation, cell cycle regulation, myocyte and adipocyte differentiation [10]. It has been partially proved that lncRNAs regulate the differentiation of ESCs and iPSCs. For instance, lncRNA Gas5 has an important role in maintaining the self-renewal and pluripotency of mESCs and iPSC [11]. Previous findings elucidated the roles of linc1281 and its m6A modification in ESCs differentiation [12]. However, the roles of lncRNA in the differentiation of ESCs into pacemaker-like cells are still unclear. Therefore, the present study aims to explore the roles of lncRNAs in the differentiation of ESCs into pacemaker-like cells.

In this study, we detected the expression of lncRNAs in the differentiated ESCs at day 6 + 5 and day 6 + 10 by high-throughput sequencing and found that the expression level of lncRNA NONMMUG016054.2 was significantly decreased, and the expression level of lncRNA was time-correlated with the process of heart development. Therefore, it was speculated that this gene might play an important role in the process of heart development, and we named it “LncRNA RCPCD” (Regulator of Cardiac Pacemaker Cell Differentiation). Then, we demonstrated that RCPCD inhibited the differentiation of ESCs into pacemaker-like cells. In addition, we confirmed that RCPCD inhibited the expression of HCN4 by increasing methylation at the HCN4 promoter region. Rescue experiments confirmed that RCPCD inhibited the differentiation of ESCs into pacemaker-like cells by inhibiting the expression of HCN4. Our results confirm the role and mechanism of lncRNA RCPCD in the differentiation of ESCs into pacemaker-like cells, which could pave the path for the development of a cell-based biological pacemaker and clinical treatment of SSS diseases.

## Methods

### Cell culture

The mouse ESCs were purchased from National Cell Resource Center (Shanghai, China) and cultured in Knockout Dulbecco’s modified Eagle’s medium (DMEM) supplemented with 15% knockout Serum Replacement, 2 nM l-glutamine, 50 units/ml penicillin, 50 μg/ml streptomycin, 1% minimal essential medium nonessential amino acids, and β-mercaptoethanol, which are purchased from Thermo-Fisher and Sigma Company. The ESCs were cultured on confluent monolayers of mitomycin C-treated primary mouse embryonic fibroblasts (Millipore, USA) and the medium was added to purified recombinant mouse leukemia inhibitory factor (1000 units/ml, Millipore, USA) to kept undifferentiated characteristics. In addition, 293 T cells were cultured in DMEM medium with 4.5 g/L glucose (Thermo-Fisher, USA), which contains 10% fetal bovine serum (FBS, Thermo-Fisher, USA) and 1% penicillin–streptomycin (Sigma, USA). ESCs and 293 T cells were cultured in an incubator with 37 °C and 5% CO_2_.

### Cell differentiation and cell transfection

On day 1 of differentiation, the ESCs were dissociated into single cells by 0.05% trypsin-ethylenediaminetetraacetic acid and cultured in the differentiation medium. The differentiation medium is composed of Iscove’s Modified Dulbecco’s medium with glutamax, 20% FBS, 1% essential media nonessential amino acids, 1% penicillin–streptomycin, and 0.1 mM β-mercaptoethanol, which are purchased from Thermo-Fisher and Sigma Company. The single cells were cultured in hanging drops to form embryoid bodies (EBs) for 2 days. On day 3, the EBs were transferred to ultra-low-attachment dishes (Corning, USA) and transfected with adenoviral at a multiplicity of infection (MOI) of 500. Adenoviral contained the RCPCD vector, HCN4 vector, control vector, RCPCD shRNA, HCN4 shRNA, or NC shRNA, respectively. EBs were transfected with these adenoviral separately or simultaneously. On day 6, control or transfected EBs were plated onto gelatin-coated cultured dishes. The EBs were transfected again on day 6 and day 6 + 1 with the same MOI as day 3 to maximize somatic gene transfer. The cells were observed and photographed under an inverted microscope at day 6 + 1 (d1), day 6 + 5 (d5), and day 6 + 10 (d10). The cells at d1, d5, and d10 also were collected for further experiments. In the differentiation of ESCs into pacemaker-like cells, 5-azacytidine (AZA, 5 μm, Sigma, USA) was added into the differentiation medium at day 3, day 6, and cultured 48 h.

### Flow cytometry

EBs were dissociated into single cells and centrifuged at 1000 × *g* for 5 min at room temperature. The cell density was ~10^6^/ml. ESCs were first stained with an anti-cardiac isoform of cTNT (1:500, rabbit monoclonal, Abcam, USA) and anti-mouse NKX2.5 (1:100, goat monoclonal, Abcam, USA) for 1 h at 37 °C. Following, the cells were stained with goat-anti-rabbit IgG-PE (1:1000, Abcam, USA) and rabbit anti-goat IgG-FITC (1:100, Abcam, USA) secondary antibodies for 30 min at 37 °C. Flow cytometry analysis was performed on a FACS Caliber flow cytometer (Beckton Dickinson, USA) and analyzed with BD CellQuest Pro software (BD, USA).

### Quantitative real-time polymerase chain reaction (qRT-PCR)

Total RNA was extracted from differentiated or transfected ESCs by using Trizol reagent (Thermo-Fisher, USA). RNA was reverse transcribed to cDNA by using PrimeScript RT reagent Kit (Takara, Japan) according to the manufacturer’s protocol. RT-PCR was performed by using AceQ Universal SYBR qPCR Master Mix (Vazyme, China) according to the manufacturer’s protocol on an Applied Biosystems 7300 sequence detection system (Applied Biosystems, USA). GAPDH levels were used as the internal control. The relative expression of genes was calculated by 2^−△△Ct^. The forward primer sequence of RCPCD is 5′- AGCAGGAAACGCCAGTGCTT-3′, reverse primer sequence of RCPCD is 5′-TCCCACGCTCAGTCAGCTCT-3′. The forward primer sequence of HCN4 is 5′-GACGGCTCCTACTTTGGAGAG-3′, reverse primer sequence of HCN4 is 5′-TCCAGCACCTCATTGAAGTTG-3′. SHOX2 forward primer sequence is 5′-CTGAAAGATCGCAAAGAGGATG-3′, reverse primer sequence is 5′-ATGAAGGCGTCGGGATAGTG-3′. Tbx3 forward primer sequence is 5′-AGCCTGTTCCCTTACCCCTAC-3′, reverse primer sequence is 5′-GTTCAGAGCCCGAGTCCACT-3′. Cx45 forward primer sequence is 5′-TTAGGGTTTGGGACCATTCG-3′, reverse primer sequence is 5′-GCTGCCATACTGCTGTTCCTG-3′.

### Western blot analysis

The total proteins were extracted from treated and differentiated ESCs using RIPA lysis buffer. The protein concentration was detected by a BCA kit (Thermo-Fisher, USA). Protein samples were fractionated by sodium dodecyl sulfate-polyacrylamide gel electrophoresis gel and transferred to polyvinylidene fluoride membrane (Millipore, USA). Subsequently, the membranes were incubated with the 5% non-fat milk for 2 h at room temperature. Following, the membranes were incubated with primary antibodies at 4 °C overnight. The primary antibody including HCN4 (1:1000 dilution), SHOX2 (1:2000 dilution), Tbx3 (1:2000 dilution) and Cx45 (1:1000 dilution). After washed by phosphate buffered saline with tween 20, the members were incubated with goat-anti-rabbit secondary antibody (1:5000 dilution) at room temperature for 2 h. The members were visualized by using a chemiluminescence kit (Vazyme, China). GAPDH expression was used as the internal control for relative gene expression at protein levels. Image J was used to quantitatively analyze the protein expression.

### Immunofluorescence (IF)

Treated and differentiated ESCs were cultured in 24-well plates and grown on the cover glasses. Cells were fixed in 4% paraformaldehyde for 1 h and then permeabilized with 0.25% Triton X-100 at room temperature for 15 min. Following, the cells were blocked with goat serum at room temperature for 30 min and then incubated with primary antibody at 4 °C overnight. The primary antibody including HCN4 (1:50 dilution), SHOX2 (1:100 dilution), Tbx3 (1:100 dilution), and Cx45 (1:50 dilution) antibodies, were purchased from Abcam. After wash three times with PBS, the cells were incubated with goat-anti-rabbit IgG secondary antibody (1:500 dilution, Abcam, USA) for 1 h at room temperature. The cell nucleus was stained by DAPI. The images were captured with a fluorescence microscope (Nikon, Japan).

### High-throughput sequencing

The lncRNA expression in d5 and d10 cells were analyzed through high-throughput sequencing (Decode Genomics, China). In brief, total RNA was extracted from cells through Trizol reagent, and the RNA concentration, purity, and integrity were assessed and adjusted. Then, ribosomal RNA (rRNA) was removed by rRNA Removal Kit (Epicentre), and the remaining RNA was proposed to generate a sequencing library according to the methods as previously described [13]. Following, the library was sequenced on Illumina Hiseq X10 platform (Illumina) and the expression of lncRNAs was analyzed by Feature Counts.

### Scoring the beating of EBs

The beating number of spontaneously beating EBs was counted for 1 min under a light microscope. RCPCD overexpression or control EBs were counted at the differentiation time point during d5 to d10. RCPCD and HCN4 overexpression, RCPCD and HCN4 knockdown, RCPCD overexpression, RCPCD knockdown, and corresponding control EBs were counted at d10. Every group spontaneously beating EBs was counted three times, the beating frequency was shown as the mean ± standard deviation (SD).

### Fluorescence in situ hybridization (FISH)

FISH assay was performed according to the instructions of the Fluorescent In Situ Hybridization Kit (RiboBio, China). In brief, differentiated ESCs at d10 were cultured in 24-well plates (6 × 10^4^/well) and grown on the glass coverslips. The glasses were washed with PBS and fixed in 4% paraformaldehyde at room temperature for 10 min when the cell confluence was up to 60–70%. Then the cells were washed by PBS and permeabilized with 0.5% Triton X-100 at 4 °C for 5 min. Following, the cells were incubated with prehybridization buffer at 37 °C for 30 min, and then incubated with hybridization buffer containing RCPCD probe (The forward primer is 5′-ACTTTGGACCCTGCACAGCC-3′, reverse primer is 5′-AAGCACTGGCGTTTCCTGCT-3′) at 37 °C overnight. The cell nucleus was stained by DAPI for 10 min after washed three times by hybridization buffer and once by PBS. Then the images were captured with a fluorescence microscope (Nikon, Japan). The process needs to be performed in dark after prehybridization.

### Luciferase reporter gene assay

Wild-type HCN4 (HCN4-WT) sequences that containing the RCPCD binding sites and mutant HCN4 (HCN4-Mut) sequences that containing the mutated RCPCD binding sites were amplified and inserted into a luciferase reporter plasmid (PMIR-REPORT Luciferase). RCPCD sequences were also inserted into PGL3 vector. 293 T cells were cultured in 24-well plates and transfection was performed using Lipofectamine 2000 (Thermo-Scientific, USA) when the confluence of cells was up to 60%. 293 T cells were co-transfected with the β-galactosidase plasmid, wild-type HCN4/mutant HCN4 plasmid, and RCPCD vector/NC vector. After 48 h of transfected, cells were harvested and lysed. Then the firefly and Renilla luciferase activities were measured by using dual-luciferase reporter assay system (Promega Corporation, USA). The ratio of Firefly to Renilla luciferase activity was determined to eliminate the variation in the transfection efficiencies.

### Methylation-specific PCR (MSP)

The DNA was extracted from control EBs at d1, d5, d10, overexpressed or AZA treated EBs at d10 through phenol-chloroform. Then, DNA was bisulfite-converted using the EZ DNA Methylation kit (Zymo Research, USA) according to the manufacturer’s instructions. The unmethylated DNA converted cytosines to uracils at CpG dinucleotides and the methylated cytosines not converted. MSP primers of HCN4 were designed to specifically amplify the target regions of unmethylated CpG dinucleotides by detecting uracils and methylated CpG dinucleotides by detecting cytosines. MSP was performed by using Power Taq PCR MasterMix (Bioteck, China) according to the instructions. The primer used were as follows: F: 5′-TTGTTTATAGTTTTTTGGAAGTAGTT-3′; R:5′-CCCAAAATTTTCCTAAACCAAA -3′.

### Chromatin immunoprecipitation (ChIP)

ChIP assay was performed by using EZ-ChIP Kit (Millipore, USA) according to the manufacturer’s instructions. In brief, differentiated ESCs were collected at d10. The cells were cross-link with 1% formaldehyde for 10 min and then quenched with glycine. Cell lysates were sonicated to generate chromatin fragments and then immunoprecipitated with DNMT1, DNMT2, and DNMT3 antibodies (Abcam, USA) separately. IgG antibody (Abcam, USA) was used as the negative control. Before the immunoprecipitation, input samples were obtained as the positive control. Then, qRT-PCR was used to detect the level of RCPCD in input and immunoprecipitation samples.

### RNA immunoprecipitation (RIP)

The RIP experiments were performed to confirm the interaction between DNMT1, DNMT2, DNMT3, and RCPCD. RIP assay was used by RIP RNA-binding protein immunoprecipitation kit (Milliopore, USA) following the manufacturer’s protocol. In brief, differentiated ESCs of RCPCD overexpression or knockdown were collected at d10. Differentiated ESCs also were collected at d10 as control. Cells were lysed in RIP lysis buffer for 5 min at 4 °C. Following, cell lysates were centrifuged at 13,000 × *g* for 20 min. Then cleared supernatant was collected and incubated with Protein A sepharose beads at 4 °C overnight, which conjugated with DNMT1 antibody, DNMT2 antibody, DNMT3 antibody, or negative control IgG antibody, respectively. After incubated, RNA/beads complex was washed and immunoprecipitated RNAs were extracted. qRT-PCR was used to detect the expression of RCPCD.

### Single-cell electrophysiology

To examine the electrophysiological properties of individual cardiomyocytes derived from mESC, EBs at D6 + 10 were dispersed into single cells according to previous research with slight modifications [14]. EBs were digested with a nominally calcium-free solution containing collagenase B at 37 °C for 50 min followed by digestion with Liberase at 35 °C for 20 min. Single, whole-cell action potential (AP) and ionic current measurements were carried out using standard microelectrode whole-cell, patch-clamp technique with an Axopatch 200B amplifier (Molecular Devices) with a sampling rate of 20 kHz and low-pass Bessel-filtered at 5 kHz. Experiments were performed at 33 °C. Cells were perfused with a normal Tyrode’s solution containing NaOH. Microelectrodes had tip resistances of 2–4 MΩ when filled with the internal recording solution. Spontaneous APs were recorded with *I* = 0 mode. Effects of adrenergic and cholinergic agonists on AP firing rate were tested by perfusing cells with Tyrode solution containing isoproterenol (1 mM, Sigma) or acetylcholine (50 nM; Sigma). Data were corrected for the estimated liquid junction potentials (−12.5 mV). The diastolic depolarization rate was determined from the slope of a 20-ms segment after the maximum diastolic potential. Funny currents (If) were recorded in a voltage-clamp mode with cells bathed in normal Tyrode’s solution containing 1 mM BaCl2 to block contaminating inward rectifier K + currents (IK1). Holding potential was set at −35 mV, and a family of voltage steps from −135 mV to +35 mV for 1.5 s with 10 mV increment was applied to elicit If. The If was identified by their time-dependent activation and their sensitivity to ivabradine (10 μM; Sigma-Aldrich).

### Statistical analysis

All data were presented as the mean ± standard deviation (SD) of three independent experiments. One-way analysis of variance and Student’s *t* test were used to test the mean difference between groups. All data were analyzed using GraphPad Prism 5.0 (Graphpad, Inc.) and Image J Software. *P* value < 0.05 was considered statistically significant.

## Results

### LncRNA RCPCD involved in the differentiation of ESCs into pacemaker-like cells

**Fig. 1:**
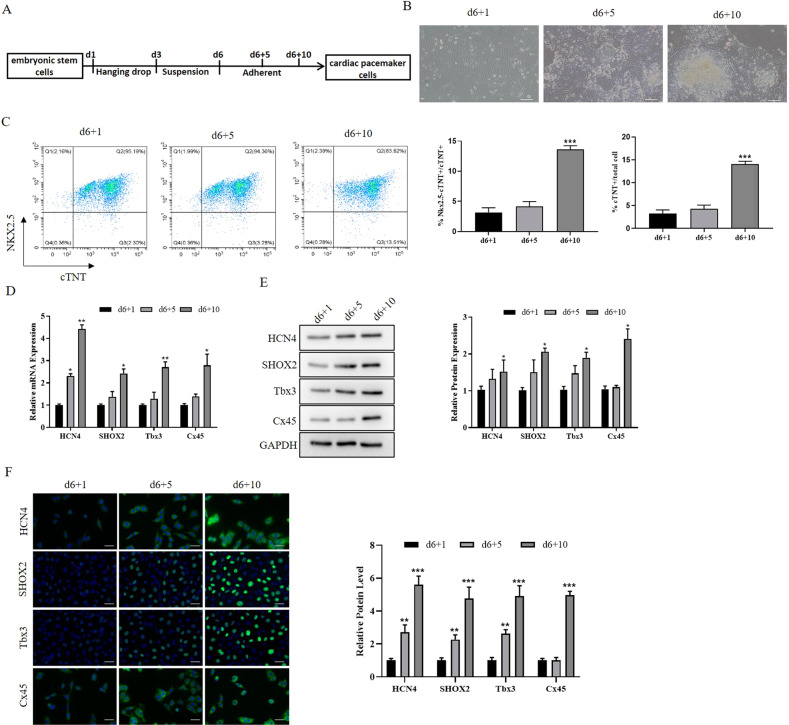
ESCs differentiated into cardiac pacemaker-like cells. **A** The diagram showing the differentiation of ESCs into pacemaker-like cells; **B** cell morphology of differentiated ESCs at d1, d5, and d10. **C** Flow cytometry detected the ratio of pacemaker-like cells at d1, d5, and d10. **D** qRT-PCR detected the expression of pacemaker-related genes at mRNA levels. **E** Western blot detected the expression of pacemaker-related genes at protein levels. **F** IF was used to detect the expression of pacemaker-related genes. *P < 0.05, **P < 0.01, * as the difference that compared with the d6 + 1 cells. Error bars were represented the mean ± SD in triplicate experiments.

To explore the lncRNAs functions in embryonic heart development of pacemaker formation, we induced ESCs differentiation to pacemaker‐like cells and detected the expression change of lncRNAs. mESCs were differentiated to form EBs by culturing them in suspension media for 6 days and then transferring them to adherent media as previously described [15]. The diagram of ESCs differentiation into pacemaker-like cells was shown in Fig. 1A. EBs were observed at d6 + 1, d6 + 5, and d6 + 10 through a microscope, and the morphology results confirmed that ESCs differentiated into pacemaker‐like cells (Fig. 1B). Pacemaker-like cells are a kind of myocardial cells with lower expression of Nxk2.5 [4]. Following, we detected the cell type through flow cytometry and the results showed that the Nxk2.5^−^ and cTNT^+^ cells were increased in the process of differentiation (Fig. 1C). Subsequently, we detected the expression of pacemaker-related genes through qRT-PCR and western blot. The qRT-PCR and western blot results showed that the expression of HCN4, SHOX2, Tbx3, and Cx45 continually increased in the differentiation process (Fig. 1D, E). Similarly, the IF results showed that HCN4, SHOX2, Tbx3, and Cx45 expression increased in the differentiation process (Fig. 1F). These results confirmed the differentiation of ESCs to pacemaker-like cells.

**Fig. 2:**
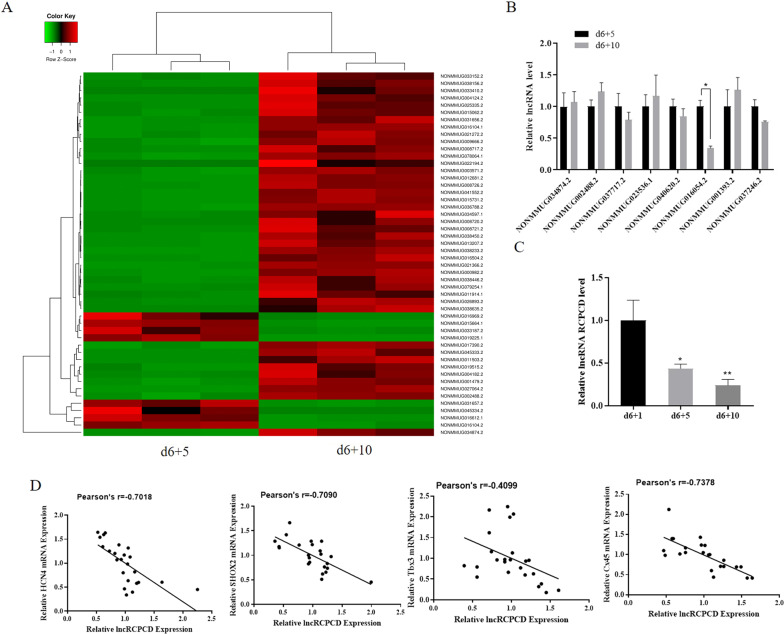
LncRNA RCPCD involved in the differentiation of ESCs into pacemaker-like cells. **A** Heat map showing the differential expression of lncRNAs in differentiated ESCs at d6 + 5 and d6 + 10; **B** qRT-PCR was used to verify the differential expression of lncRNAs. **C** qRT-PCR detected the expression of lncRNA RCPCD in the differentiated ESCs at d6 + 1, d6 + 5, and d6 + 10. **D** Correlation analysis the expression of RCPCD and pacemaker-related genes in the differentiation of ESCs into pacemaker-like cells. *P < 0.05, **P < 0.01, * as the difference that compared with the d1 cells. Error bars were represented the mean ± SD in triplicate experiments.

The different expressions of lncRNAs were detected in EBs at d6 + 5 and d6 + 10 through RNA sequencing, 50 differentially expressed lncRNAs were displayed through heatmap (Fig. 2A). qRT-PCR was used to validate the expression of lncRNAs, which discovered significant expression differences in RNA sequencing. The results showed that only lncRNA RCPCD (NONMMUG016054.2) expression was significantly downregulated in d6 + 10 EBs compared with d6 + 5 EBs (Fig. 2B). We further detected the expression of RCPCD in EBs at d6 + 1, d6 + 5, and d6 + 10. The results displayed that the expression of RCPCD was gradually decreased in the differentiation process (Fig. 2C). Correlation analysis showed that RCPCD expression negatively correlated with pacemaker-like genes (Fig. 2D). These results suggested that RCPCD may play an important role in the differentiation of ESCs into pacemaker-like cells.

### Overexpression of lncRNA RCPCD suppressed the differentiation of ESCs into pacemaker-like cells

**Fig. 3:**
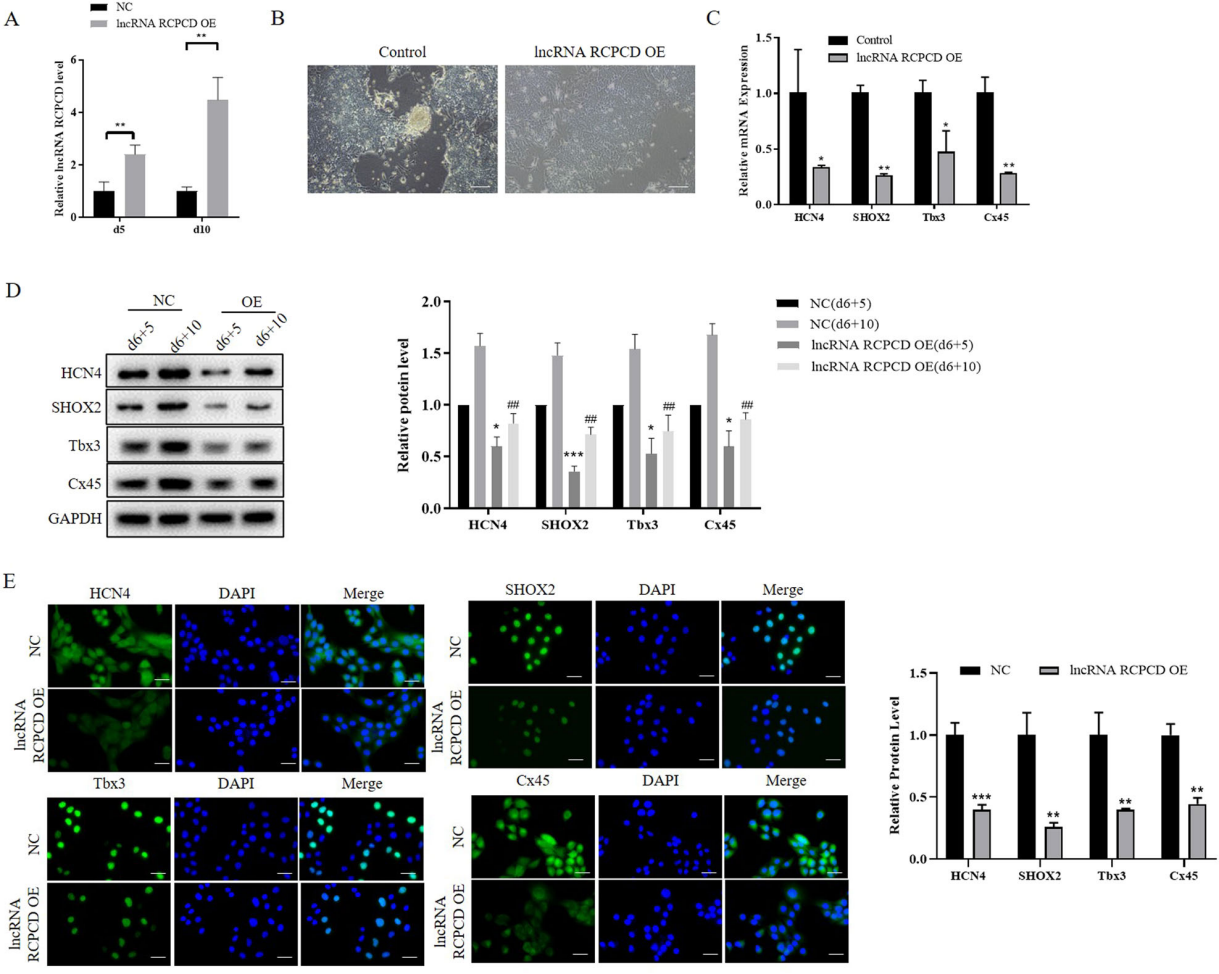
Overexpression of lncRNA RCPCD suppressed the differentiation of ESCs into pacemaker-like cells. ESCs were cultured in a differentiation medium to form pacemaker-like cells. RCPCD adenovirus and control adenovirus were transfected into ESCs when cultured 3 days and 6 days. **A** qRT-PCR detected the expression of RCPCD in differentiated ESCs at d6 + 5 and d6 + 10. **B** Cell morphology of differentiated ESCs at d6 + 10. **C** qRT-PCR detected the expression of pacemaker-related genes in differentiated ESCs at d6 + 10. **D** Western blot detected the expression of pacemaker-related genes in d6 + 5 and d6 + 10 differentiated ESCs. **E** IF was used to detect the expression of HCN4, Cx45, SHOX2, and Tbx3 in differentiated ESCs at d6 + 10. *P < 0.05, **P < 0.01, ***P < 0.01, * as the difference that compared with the control group ESCs at d6 + 5 and d6 + 10. ^##^*P* < 0.01, ^#^ as the protein levels difference that compared with control group ESCs at d6 + 10. Error bars were represented the mean ± SD in triplicate experiments.

To explore the roles of lncRNA RCPCD in the differentiation of ESCs into pacemaker-like cells, we transfected RCPCD vector adenovirus into ESCs as the previous description. The d6 + 5 and d6 + 10 EBs were collected and the expression of RCPCD was detected by qRT-PCR. The results showed that RCPCD expression was increased in RCPCD transfected EBs compared with control EBs. And the expression of RCPCD was significantly increased in RCPCD transfected EBs at d6 + 5 and d6 + 10 (Fig. 3A). Following, we detected the differentiation change of EBs at d6 + 10. Cell morphology was observed through microscopic and the results showed overexpression of RCPCD inhibited the differentiation of ESCs to pacemaker‐like cells (Fig. 3B). The HCN4, SHOX2, Tbx3, and Cx45 expression was detected by qRT-PCR and the results showed that the expression levels of HCN4, Cx45, SHOX2, and Tbx3 were significantly decreased in the RCPCD overexpression EBs compared with NC EBs (Fig. 3C). These gene expressions at protein levels were detected by western blot in d6 + 5 and d6 + 10 EBs (Fig. 3D). The results also showed that HCN4, Cx45, SHOX2, and Tbx3 expression were decreased in RCPCD overexpression EBs at d5 and d10 (Fig. 3D). Consistently, the IF results showed that RCPCD decreased the expression of HCN4, Cx45, SHOX2, and Tbx3 in d10 EBs (Fig. 3E).

**Fig. 4:**
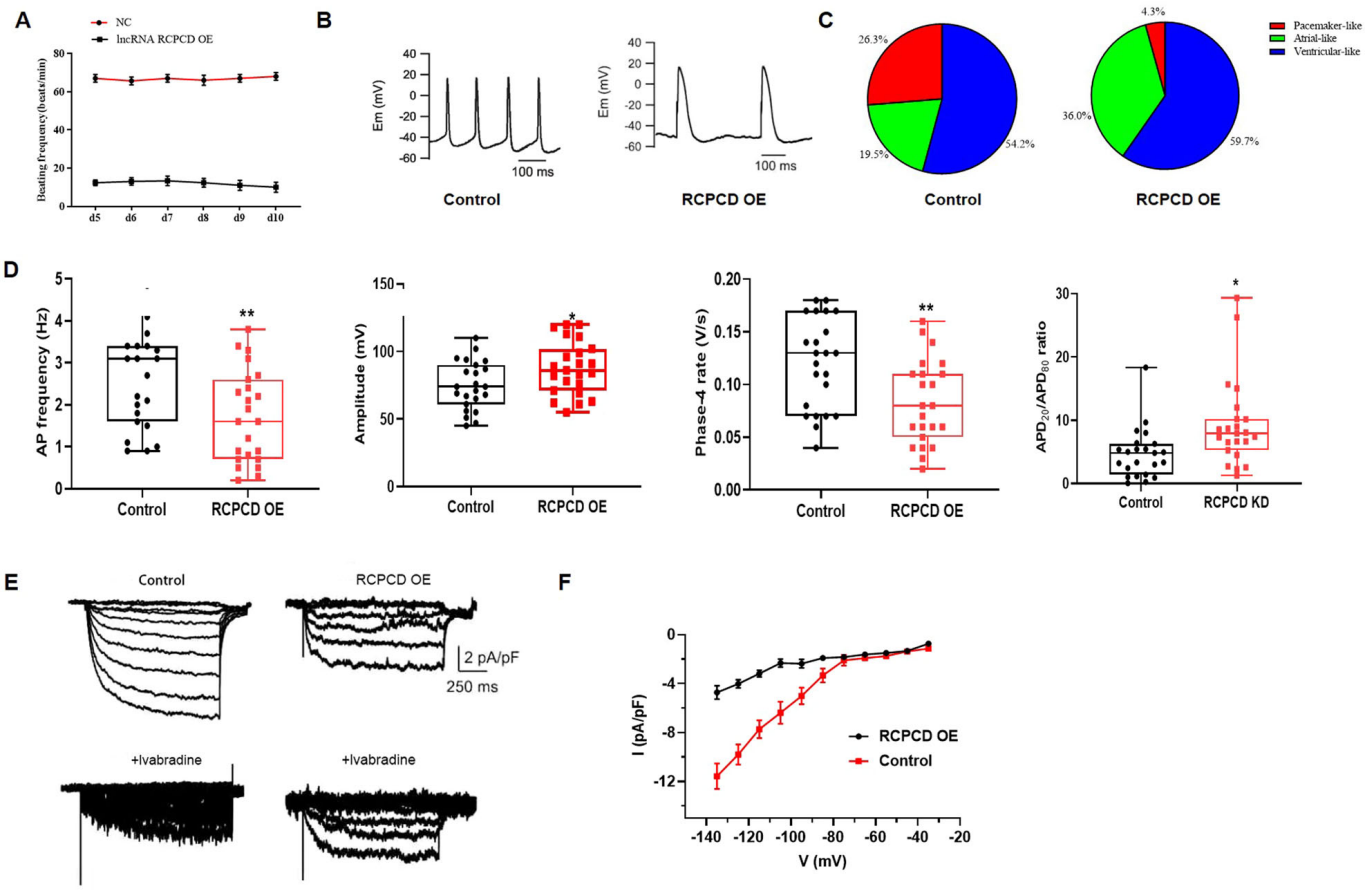
LncRNA RCPCD-EBs exhibited pacemaker cell-like electrophysiology. **A** Beating frequency was detected at different time points during the differentiation of embryonic stem cells into pacemaker cells. **B** Representative raw traces of APs recorded from spontaneously beating single cells from control EBs (left) or RCPCD Overexpression EBs (right). **C** The ratio of pacemaker-like cells at d6 + 10. **D** Comparison of AP parameters (from left to right): spontaneous beating rate, the amplitude of the AP upstroke, spontaneous phase 4 diastolic depolarization rate, and the ratio of AP duration at 20% repolarization to 80% repolarization. **E** Representative HCN ionic currents (If) recorded from a control EB cell (left) and an RCPCD Overexpression EB cell (right). Lower panels show the inhibition of time-dependent currents upon the addition of 10 mM ivabradine in the bath solution. **F** Current density-voltage relationships of control and RCPCD overexpression groups. *P < 0.05, **P < 0.01, ***P < 0.01, * as the difference that compared with the control group.

We further evaluated the effect of RCPCD on pacemaker-like cell differentiation through electrophysiology. we characterized APs from freshly isolated, spontaneously beating cells from RCPCD overexpression EBs or control EBs. We detected the beating frequency of NC EBs and RCPCD overexpression EBs at different time points during the differentiation. The results showed that overexpression RCPCD significantly inhibited the beating frequency of EBs (Fig. 4A). The single cells isolated from control EBs exhibited more ratio of pacemaker-like AP profile than the single cells isolated from RCPCD overexpression EBs (Fig. 4B, C). Single cells from RCPCD overexpression EBs also fired APs at a lower rate than control cells (Fig. 4D, left). The mean amplitude of spontaneous APs was higher in RCPCD overexpression EBs cells (Fig. 4D, second from left). Action potential duration was longer, and each AP was preceded by slower phase 4 depolarization in RCPCD overexpression EBs cells compared with control (Fig. 4D, right two panels). The density of If was significantly higher in RCPCD knockdown EBs cells compared with control (Fig. 4E, F). Thus, control EBs cells exhibited more pacemaker-like cells AP parameters than the RCPCD overexpression EBs cells. Moreover, in RCPCD knockdown EBs, the pacemaker-like cells showed an obviously increased ratio, as indicated in Supplementary Fig. 1, suggesting that the lncRNA RCPCD specifically blocked pacemaker differentiation other than promoting atrial differentiation. These results demonstrated that RCPCD inhibited the differentiation of ESCs into pacemaker-like cells.

### LncRNA RCPCD inhibited the expression of HCN4 by regulating the methylation of the HCN4 promoter region

**Fig. 5:**
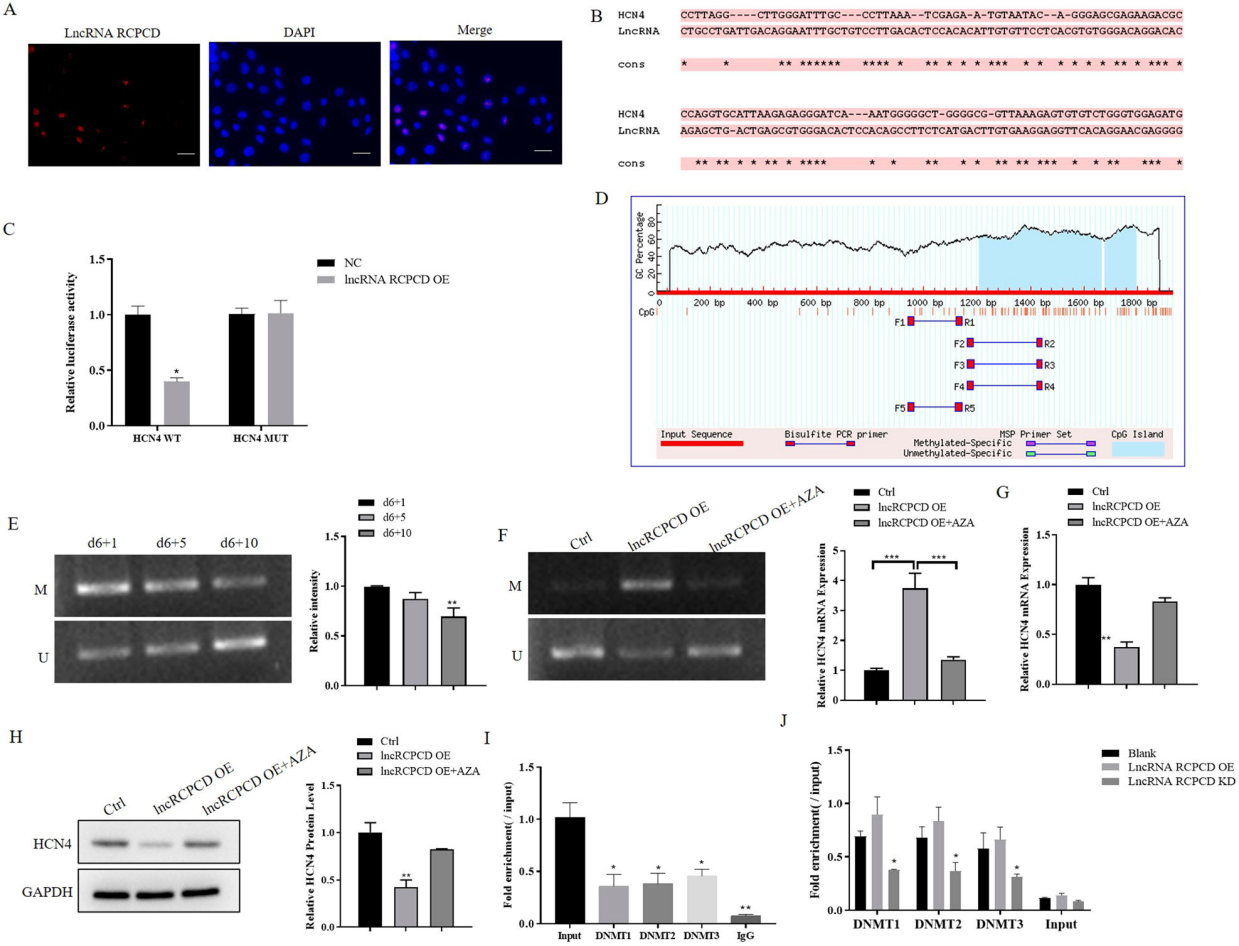
LncRNA RCPCD inhibited the expression of HCN4 by regulating the methylation of HCN4 promoter region. ESCs were cultured in a differentiation medium to form pacemaker-like cells. RCPCD adenovirus was transfected into differentiated ESCs when cultured 3 days and 6 days. In addition, AZA was added into the differentiated medium at day 3, day 6, and cultured 48 h. **A** FISH assay verified the localization of RCPCD in differentiated ESCs at d6 + 10. **B** RCPCD and HCN4 binding sites. **C** Luciferase reporter gene assay was used to confirm the target binding of RCPCD and HCN4 in 293 T cells. **D** Methprimer predicted the distribution of CpG island in the HCN4 gene promoter region. **E** MSP assay detected methylation levels in d6 + 1, d6 + 5, and d6 + 10 differentiated ESCs. **F** Methylation levels in control, RCPCD transfected, RCPCD transfected and AZA treated group differentiated ESCs at d6 + 10 were detected by MSP assay. **G** qRT-PCR detected the expression of HCN4 in control, RCPCD transfected, RCPCD transfected and AZA treated group differentiated ESCs at d6 + 10. **H** Western blot detected the expression of HCN4 in control, RCPCD transfected, RCPCD transfected and AZA treated group differentiated ESCs at d6 + 10. **I** The expression of RCPCD, which interacts with DNMTs, was detected by CHIP-qRT-PCR in differentiated ESCs at d6 + 10. **J** The expression of RCPCD, which interacts with DNMTs, was detected by RIP-qRT-PCR in RCPCD overexpression or knockdown differentiated ESCs at d6 + 10. *P < 0.05, **P < 0.01, * as the difference that compared with the control group or input samples. Error bars were represented the mean ± SD in triplicate experiments.

We next sought to elucidate the molecular mechanism of lncRNA RCPCD suppressed the differentiation of ESCs into pacemaker-like cells. LncRNA may regulate the gene expression through competing endogenous RNAs (ceRNA) mechanisms in the cytoplasm, while lncRNA regulates the transcription of genes through epigenetic regulation when located in the nucleus [16]. So, we detected the location of RCPCD. The FISH results showed that RCPCD mainly localized in the nucleus in d6 + 5 EBs (Supplementary Fig. 2A) and in d6 + 10 EBs (Fig. 5A). Therefore, we focus on the potential roles of RCPCD in epigenetic regulation. By biological prediction, we found that RCPCD bind to the promoter region of HCN4 (Fig. 5B). To verify the target binding, a luciferase reporter gene assay was performed. The results showed that luciferase activity was significantly decreased in HCN4-WT cells when transfected RCPCD vector. Although the luciferase activity had no significant change in HCN4-Mut cells (Fig. 5C). Subsequently, we examined the CpG island in the genomic sequence of the HCN4 promoter region through Methprimer, and found that a CpG island was located in the HCN4 promoter region (Fig. 5D), suggesting that DNA methylation may involve in the regulation of HCN4 expression. Next, we detected the DNA methylation status of HCN4 in differentiated ESCs through MSP assay. The results showed that the methylation level of the HCN4 promoter region was decreased in the progress of ESCs differentiation into pacemaker-like cells (Fig. 5E). Then, DNA methylation of HCN4 was detected in RCPCD overexpression EBs at d10, which was treated or not treated with DNA methylation inhibitor 5-AZA. The MSP results showed that RCPCD increased the DNA methylation level of the HCN4 promoter region and the increase was inhibited by AZA (Fig. 5F). qRT-PCR and western blot results showed that RCPCD inhibited the expression of HCN4 which was restored by AZA (Fig. 5G, H). These results suggesting that RCPCD inhibited the expression of HCN4 by increasing the methylation at the promoter region.

As well known, DNA methyltransferases (DNMTs), including DNMT1, DNMT2, and DNMT3, regulate the DNA methylation at the CpG islands [17]. Therefore, to better understanding the mechanism of RCPCD regulated the HCN4 methylation, we detected the binding of DNMTs and RCPCD through ChIP assay. The results showed that RCPCD expression was increased in DNMT1, DNMT2, and DNMT3 antibody-derived samples than IgG samples, which suggesting RCPCD interacted with DNMT1, DNMT2, and DNMT3 in d6 + 5 EBs (Supplementary Fig. 2B) and in d6 + 10 EBs (Fig. 5I). In addition, to further verify RCPCD interacted with DNMTs, RIP assay was performed in RCPCD overexpression or knockdown EBs. The results showed that RCPCD binding with DNMT1, DNMT2, and DNMT3, the RCPCD binding by DNMTs was increased in d6 + 5 RCPCD overexpression EBs (Supplementary Fig. 2C) and in d6 + 10 RCPCD overexpression EBs (Fig. 5J). These results suggested that RCPCD inhibited the expression of HCN4 through DNMT-mediated DNA methylation at the promoter region.

### LncRNA RCPCD inhibited differentiation of ESCs into pacemaker-like cells by inhibiting the expression of HCN4

**Fig. 6:**
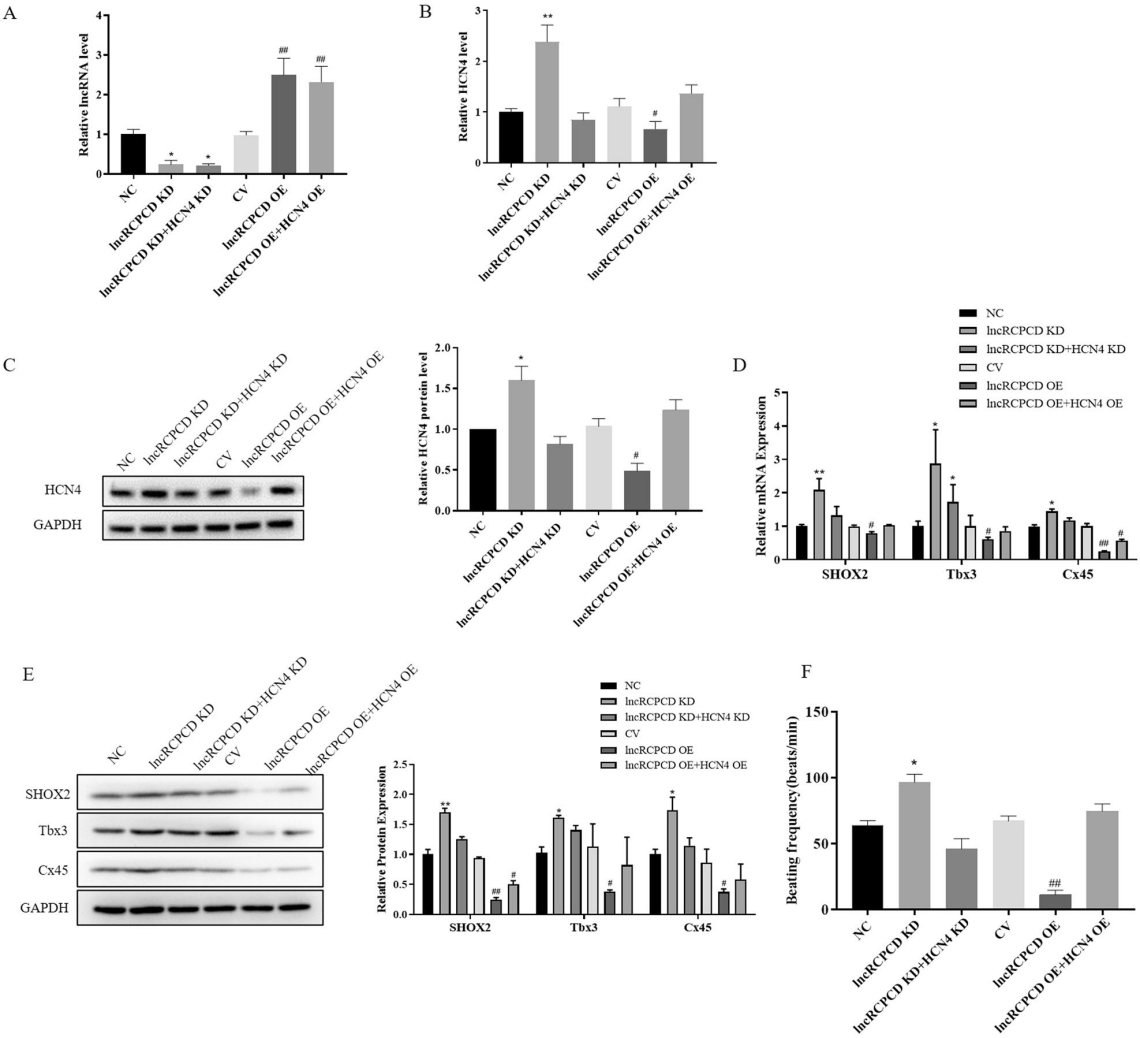
LncRNA RCPCD inhibited differentiation of ESCs into pacemaker-like cells by inhibiting the expression of HCN4. ESCs were cultured in a differentiation medium to form pacemaker-like cells. At cultured 3 days, ESCs divided into six groups and transfected adenovirus: negative control shRNA adenovirus, RCPCD shRNA adenovirus, RCPCD shRNA adenovirus, and HCN4 shRNA adenovirus, control vector adenovirus, RCPCD vector adenovirus, RCPCD vector adenovirus, and HCN4 vector adenovirus. At cultured 6 days, the same transfection was performed again. These group differentiated ESCs were collected and detected at d6 + 10. **A** qRT-PCR detected the expression of RCPCD. **B** qRT-PCR detected the expression of HCN4 at mRNA levels. **C** The expression of HCN4 at protein levels was detected by western blot. **D** qRT-PCR detected the expression of Tbx3, Cx45, and SHOX2 at mRNA levels. **E** The expression of Tbx3, Cx45, and SHOX2 at protein levels were detected by western blot. **F** Beating frequency was counted in these differentiated ESCs at d6 + 10. *P < 0.05, **P < 0.01, * as the difference that compared with negative control transfected group. ^#^*P* < 0.05, ^##^*P* < 0.01, ^#^ as the difference that compared with control vector-transfected group. Error bars were represented the mean ± SD in triplicate experiments.

To confirm lncRNA RCPCD regulated the differentiation of ESCs into pacemaker-like cells through HCN4, RCPCD shRNA adenovirus, RCPCD shRNA adenovirus, and HCN4 shRNA adenovirus, RCPCD vector adenovirus, or RCPCD vector adenovirus and HCN4 vector adenovirus were transfected into EBs separately. NC shRNA adenovirus or control vector adenovirus was transfected into EBs as the negative control. Subsequently, these EBs were collected at d6 + 10. The expression of RCPCD was detected by qRT-PCR. The results showed that RCPCD expression decreased in RCPCD shRNA-transfected EBs and the expression of RCPCD was significantly increased in RCPCD vector-transfected EBs (Fig. 6A). The expression of HCN4 at mRNA levels and protein levels were detected by qRT-PCR and western blot, respectively. The results showed that the expression of HCN4 was significantly increased in RCPCD shRNA-transfected EBs and the increase was inhibited by transferring HCN4 shRNA. Similarly, the expression of HCN4 was significantly decreased in RCPCD vector-transfected EBs and the downregulation was inhibited by transferring the HCN4 vector (Fig. 6B, C). Consistently, the IF results showed that downregulation of RCPCD could increase the expression of HCN4 and the expression was restored by transferring HCN4 shRNA. Upregulate the expression of RCPCD could inhibit the expression of HCN4 and the inhibition was attenuated by transferring HCN4 vector (Supplementary Fig. 3A). In addition, the expression of pacemaker-like cell marker genes, including SHOX2, TBX3, and Cx45, were detected by qRT-PCR, western blot, and IF. These results showed that RCPCD reduced the expression of SHOX2, TBX3, and Cx45, and the effect of RCPCD was restored by HCN4 (Fig. 6D, E and Supplementary Fig. 3B). Following, we detected the beating frequency of these EBs at d6 + 10. The results showed that knockdown of RCPCD increased the beating frequency of EBs and the increase was inhibited by downregulating the expression of HCN4. Overexpression of RCPCD significantly inhibited the beating frequency of EBs and the inhibition was retarded by HCN4 (Fig. 6F). These results demonstrated that RCPCD inhibited the differentiation of ESCs into pacemaker-like cells by inhibiting the expression of HCN4.

## Discussion

The roles of lncRNAs in maintain ESCs pluripotency and regulate cell fate have been demonstrated [18]. For instance, lncRNA H19 suppresses the differentiation of Th17 cells and proliferation of ESCs through miR-342-3p/IER3 pathway [19]. Linc1557 inhibits the differentiation of ESCs by regulating the phosphorylation levels of STAT3 and affecting its stability [20]. However, the roles and mechanisms of lncRNAs regulate the differentiation of ESCs into pacemaker-like cells is still unclear. To explore the functions of lncRNAs in the differentiation of ESCs into pacemaker-like cells, ESCs were cultured in pacemaker differentiation medium and high-throughput sequencing of differentiated ESCs was performed. The results showed that lncRNA RCPCD expression was decreased in d6 + 10 differentiated ESCs than d6 + 5 cells. Little studies have reported the roles of lncRNA RCPCD currently. To explore the roles of lncRNA RCPCD in the differentiation of ESCs into pacemaker-like cells, RCPCD adenovirus was transfected into differentiated ESCs to overexpression RCPCD. According to the cell morphology, flow cytometry, qRT-PCR, western blot, and IF results, we found that overexpression of RCPCD inhibited the differentiation of ESCs into pacemaker cells.

Many lncRNAs play their roles in the mammalian transcriptome via multiple mechanisms. Such as, linc-ROR promotes osteogenic differentiation of mesenchymal stem cells through competing for binding miR-138 and miR-145, then increases the expression of ZEB2 and actives wnt/β-catenin pathway [21]. mESC-specific linc1281 is necessary for mESC differentiation and the roles rely on m6A enrichment within the last exon of linc1281. M6A modification of linc1281 regulates mESC differentiation through ceRNA mechanisms [12]. H19 knockdown actives S-adenosyl homocysteine hydrolase, then increases DNMT3b-mediated methylation of lncRNA-coding gene Nctc1 within the lgf2-H19-Nctc1 locus [22]. LncRNAs play different roles while located in the cytoplasm or nucleus [16]. So, we detected lncRNA RCPCD location in differentiated ESCs at d6 + 10 through FISH assay and the results showed that RCPCD located in the nucleus. Therefore, we put forward hypotheses that lncRNA RCPCD inhibits the differentiation of ESCs into pacemaker-like cells through epigenetic regulation.

Pacemaker cells are characterized by abundant expression of hyperpolarization-activated cyclic nucleotide-gated potassium channel 4 (HCN4), which is the basis of funny current (If) and a key factor in the generation of regular sinus rhythm [23]. Alicia et al. [24] found that miR-423-5p regulates the expression of HCN4 and contributes to training-induced bradycardia. Yang et al. [25] found that loss of thioredoxin-2 reduces HCN4 expression via mitochondrial ROS-HDAC4-MEF2C pathway and induces SSS in mice. However, the regulation of HCN4 expression is still larger unknown. The predicted results showed that RCPCD target binding with HCN4 promoter region and the predicted results were confirmed by luciferase reporter assay. In addition, the HCN4 promoter region has the methylation site in CpG island. So, we supposed whether lncRNA RCPCD regulates the methylation of HCN4 at the promoter region and then regulates the expression of HCN4. To confirm the hypothesis, 5-AZA, a DNA methyltransferase inhibitor, was added into the differentiation medium to inhibit the methylation level. MSP results showed that RCPCD increased the methylation of HCN4 and the increase was inhibited by AZA. qRT-PCR and western blot results showed that RCPCD inhibited the expression of HCN4 at mRNA and protein levels, while AZA treatment could restore the expression of HCN4. These results suggested that RCPCD inhibited the expression of HCN4 by increasing the methylation at promoter regions.

DNMTs, including DNMT1, DNMT2, DNMT3a, and DNMT3b, regulate the DNA methylation at the CpG islands has been reported [26]. It has also been reported that lncRNAs regulated DNA methylation through interacting with DNMTs [27]. For instance, high expression of lncRNA lung cancer-associated transcript 1 in esophageal squamous cell carcinoma upregulates DNMT protein levels, thereby affecting gene transcription [28]. LncRNA HOXA11-AS promotes the proliferation and invasion of gastric cancer through the chromatin modification factors, which including polycomb repressive complex 2 and Dnmt1 [29]. Therefore, to detect RCPCD regulated the methylation of HCN4 through DNMTs, CHIP and RIP assay were performed and the results showed that DNMTs, including DNMT1, DNMT2, DNMT3, binding with RCPCD. In addition, RIP assay results also showed that DNMTs recruited by RCPCD increased in RCPCD overexpression cells. These results suggested that RCPCD increasing the methylation of HCN4 at promoter regions through recruiting DNMTs. Finally, rescues experiments were performed and the results confirmed that RCPCD inhibited the differentiation of ESCs into pacemaker-like cells by inhibiting the expression of HCN4.

In conclusion, our study clarified the role and mechanism of lncRNA RCPCD in the differentiation of ESCs into pacemaker-like cells. LncRNA RCPCD inhibited the expression of HCN4 by recruiting DNMTs and increasing the methylation level of HCN4 at the promoter region. LncRNA RCPCD inhibited the differentiation of ESCs into pacemaker-like cells by inhibiting the expression of HCN4. This study could pave the path for the development of a cell-based biological pacemaker and may promote the therapy of SSS diseases.

## Supplementary information

Supplementary legends

Supplementary Figure1

Supplementary Figure 2

Supplementary Figure3
