# Supplementary material for: Downregulated lncRNA RCPCD promotes differentiation of embryonic stem cells into cardiac pacemaker-like cells by suppressing HCN4 promoter methylation
Source: Cell Death Dis. 2021 Jul 2;12(7):667. doi: 10.1038/s41419-021-03949-5 (PMC8253811; doi:10.1038/s41419-021-03949-5)
Supplement: Supplementary file 1 — Supplementary legends [file 41419_2021_3949_MOESM1_ESM.docx]

**Supplementary figure 1.** **Knockdown of lncRNA RCPCD inhibited pacemaker cell-like differentiation of ESCs.**

APs from freshly isolated, spontaneously beating cells from Control-EBs or RCPCD knockdown-EBs were characterized and the ratio of pacemaker-like cells were shown.

**Supplementary figure 2.** **LncRNA RCPCD inhibited the expression of HCN4 by regulating the methylation of HCN4 promoter region.**

ESCs were cultured in differentiation medium to form pacemaker-like cells. RCPCD adenovirus were transfected into differentiated ESCs when cultured 3 days and 6 days. Additionally, AZA was added into differentiated medium at day 3, day 6 and cultured 48 h. (A) FISH assay verified the localization of RCPCD in differentiated ESCs at d6+5. (B) The expression of RCPCD, which interact with DNMTs, was detected by CHIP-qRT-PCR in differentiated ESCs at d6+5. (J) The expression of RCPCD, which interact with DNMTs, was detected by RIP-qRT-PCR in RCPCD overexpression or knockdown differentiated ESCs at d6+5. **P* < 0.05, ***P* < 0.01, * as the difference that compared with the control group or input samples. Error bars were represented the mean ± SD in triplicate experiments.

**Supplementary figure 3.** **LncRNA RCPCD inhibited differentiation of ESCs into pacemaker-like cells through inhibiting the expression of HCN4.**

ESCs were cultured in differentiation medium to form pacemaker-like cells. At day 3, ESCs divided into six group and transfected adenovirus: negative control shRNA adenovirus, RCPCD shRNA adenovirus, RCPCD shRNA adenovirus and HCN4 shRNA adenovirus, control vector adenovirus, RCPCD vector adenovirus, RCPCD vector adenovirus and HCN4 vector adenovirus. At day 6, the same transfection was performed again. These group differentiated ESCs were collected and detected at d6+10. (A) IF was used to detect the expression of HCN4. (B) IF was used to detect the expression of Tbx3, Cx45 and SHOX2.
