# Supplementary figures and images for: Downregulated lncRNA RCPCD promotes differentiation of embryonic stem cells into cardiac pacemaker-like cells by suppressing HCN4 promoter methylation
Source: Cell Death Dis. 2021 Jul 2;12(7):667. doi: 10.1038/s41419-021-03949-5 (PMC8253811; doi:10.1038/s41419-021-03949-5)

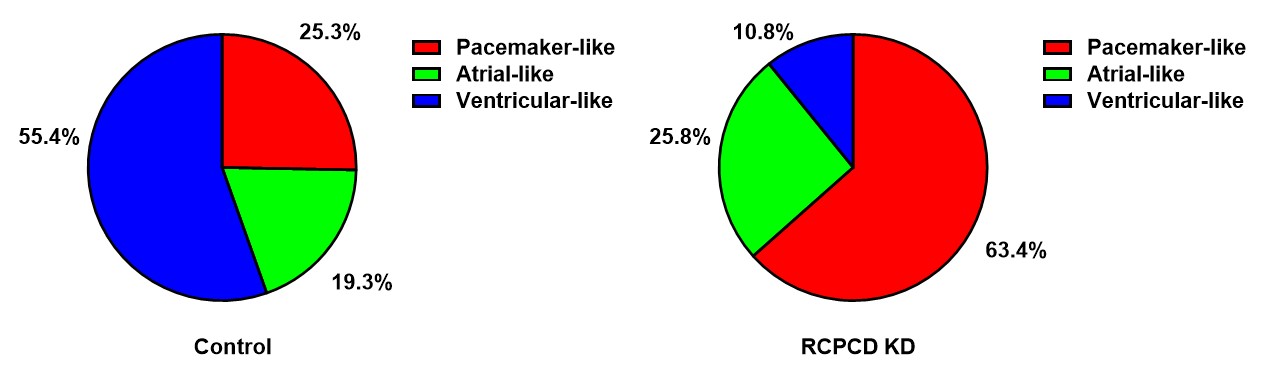

Supplement: Supplementary file 2 — Supplementary Figure1 [file 41419_2021_3949_MOESM2_ESM.jpg]

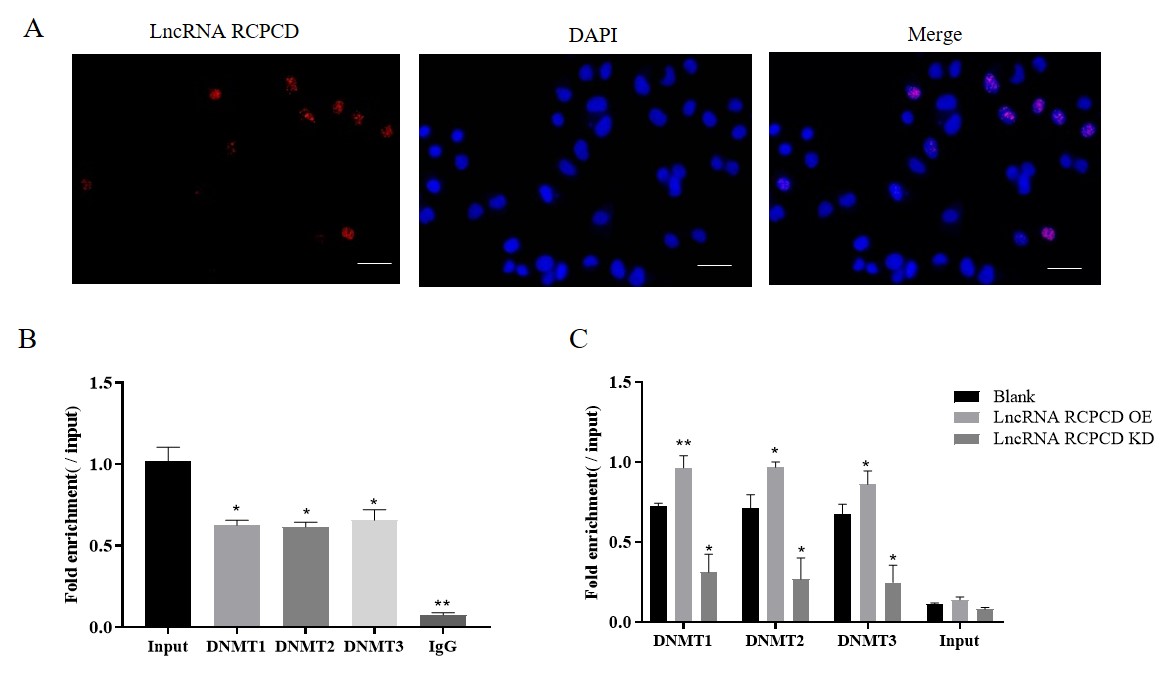

Supplement: Supplementary file 3 — Supplementary Figure 2 [file 41419_2021_3949_MOESM3_ESM.jpg]

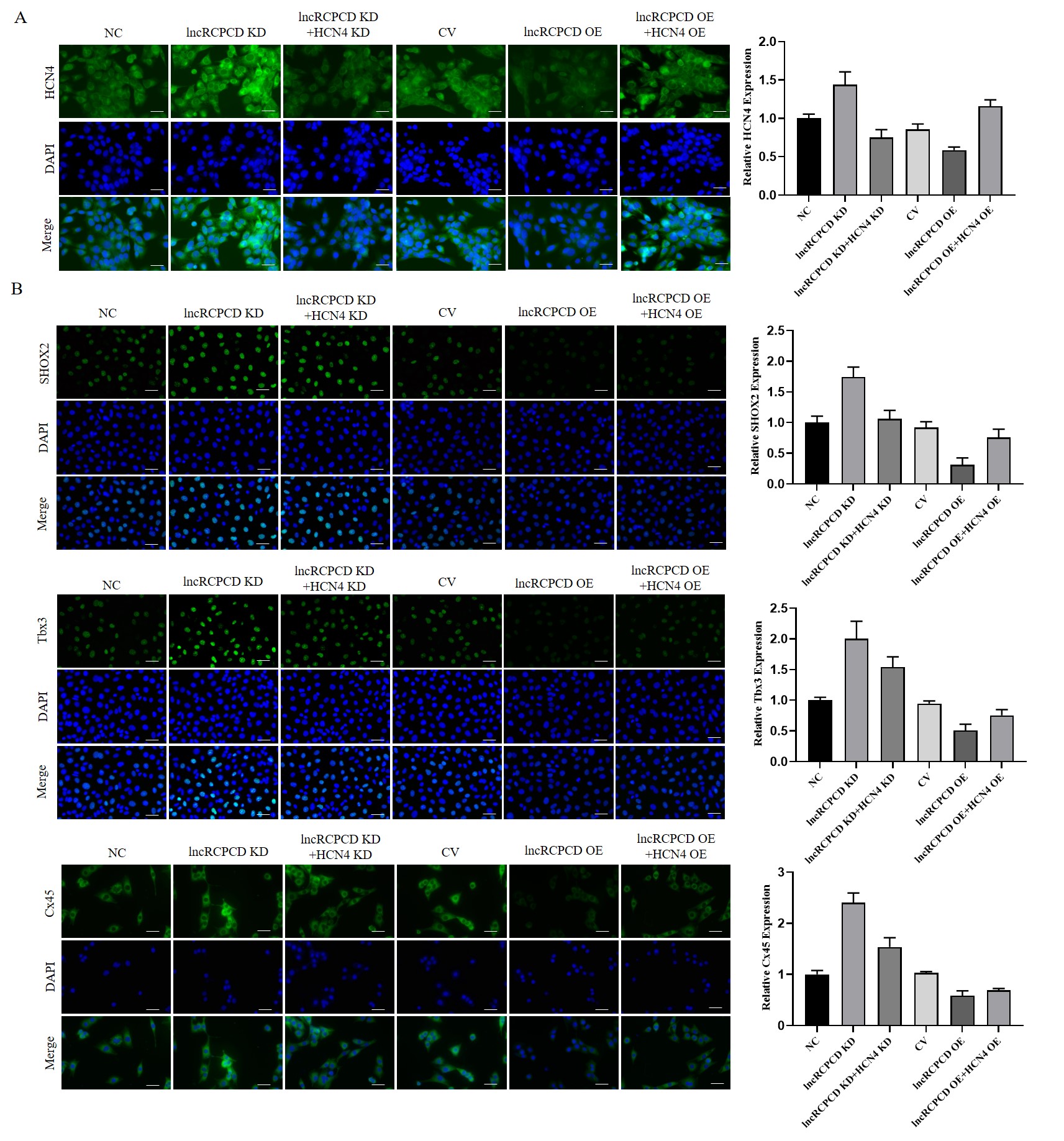

Supplement: Supplementary file 4 — Supplementary Figure3 [file 41419_2021_3949_MOESM4_ESM.jpg]
